# Microplastic in riverine fish is connected to species traits

**DOI:** 10.1038/s41598-018-29980-9

**Published:** 2018-08-03

**Authors:** R. E. McNeish, L. H. Kim, H. A. Barrett, S. A. Mason, J. J. Kelly, T. J. Hoellein

**Affiliations:** 1 Loyola University Chicago – Biology, 1032 West Sheridan Road, Chicago, IL 60660 USA; 2 Department of Geology and Environmental Sciences, The State University of New York at Fredonia, 280 Central Ave., Science Complex 340, Fredonia, NY 14063 USA

## Abstract

Microplastic is a contaminant of concern worldwide. Rivers are implicated as major pathways of microplastic transport to marine and lake ecosystems, and microplastic ingestion by freshwater biota is a risk associated with microplastic contamination, but there is little research on microplastic ecology within freshwater ecosystems. Microplastic uptake by fish is likely affected by environmental microplastic abundance and aspects of fish ecology, but these relationships have rarely been addressed. We measured the abundance and composition of microplastic in fish and surface waters from 3 major tributaries of Lake Michigan, USA. Microplastic was detected in fish and surface waters from all 3 sites, but there was no correlation between microplastic concentrations in fish and surface waters. Rather, there was a significant effect of functional feeding group on microplastic concentration in fish. *Neogobius melanostomus* (round goby, a zoobenthivore) had the highest concentration of gut microplastic (19 particles fish^−1^) compared to 10 other fish taxa measured, and had a positive linear relationship between body size and number of microplastic particles. Surface water microplastic concentrations were lowest in the most northern, forested watershed, and highest in the most southern, agriculturally dominated watershed. Results suggest microplastic pollution is common in river food webs and is connected to species feeding characteristics. Future research should focus on understanding the movement of microplastic from point-source and diffuse sources and into aquatic ecosystems, which will support pollution management efforts on inland waters.

## Introduction

In the mid-1900s, plastic became an integral component of human cultures and commerce globally^1^. Plastic contributes to ~10% of all municipal waste^2^ and 50–80% of waste on beaches and in the oceans^3^. Plastic litter is an emerging concern in ecosystems worldwide. Approximately 20 million tons of plastic enters the marine environment each year^4^, and plastic litter is predicted to outweigh fish in the ocean by the year 2050^5^. Plastic is abundant in the most remote, un-inhabited parts of the world such as the Barents Sea (Artic Ocean)^6^, Henderson Island (South Pacific)^7^, and the deep ocean^8,9^. Sources of plastic litter to aquatic ecosystems include wastewater treatment plant effluent^10,11^, industrial production^12^, synthetic textiles^13^, and the breakdown of larger anthropogenic litter (AL; trash) into smaller pieces^14–16^. While a growing body of research shows plastic is ubiquitous globally, its biological and ecological effects are less well known.

Microplastic (particles <5 mm) is a focus of research on interactions between plastic and biota, including microbes, invertebrates, fish, birds, and aquatic mammals^17–20^. Microplastic can adsorb hydrophobic compounds such as persistent organic pollutants and contaminants of emerging concern (*e.g*. Triclosan and polyaromatic hydrocarbons (PAHs))^21–23^. Once ingested, compounds can be desorbed in the anaerobic environment of the gut and absorbed by animal tissues^21,23^. This may accelerate bioaccumulation of microplastic and adsorbed compounds as they move through food webs via trophic transfer^24^. For example, up to 60 ng g^−1^ dry weight of pyrene (a PAH) was measured in the gill tissue of a filter-feeding mussel (*Mytilus galloprovincialis* Lamarck) after consuming microplastic exposed to pyrene^22^. Ingestion of microplastic could also reduce nutrient assimilation via digestive tract blockages and irritation of epithelial lining^25,26^. Finally, microplastic supports unique microbial communities compared to natural habitats and substrates^20,27^ and could facilitate pathogenic ‘hitch-hikers’ such as the bacteria Campylobacteraceae and *Vibrio*^10,19,20^.

Although microplastic ecology is a rapidly growing field of research, most studies have focused on marine organisms and habitats, with fewer studies conducted in rivers. Understanding the abundance, movement, and biological interactions of microplastic in freshwaters is critical to documenting its global impacts^28^. In addition, because freshwater ecosystems are closely connected to the terrestrial landscape and have much smaller volumes of water than the oceans, freshwaters represent important sites for prevention and management of microplastic.

Landscape features in a watershed (*e.g*., land-use, riparian vegetation, and geomorphology) influence particle transport and concentration in rivers^29–33^, but few studies have examined how landscape features influence the abundance and biological interactions of microplastic in freshwaters. Microplastic originates from point and non-point sources. Earlier studies have focused on point sources of microplastic pollution such as WWTP effluent^10,34,35^, while non-point sources of pollution are less well understood. Recent evidence suggests non-point sources include application of biosolids to agricultural fields^36^, atmospheric deposition^37^, and stormwater runoff^38^. Landscape features that serve as sinks for other types of fine particles most likely promote deposition of microplastic, such as dams, lakes, and low-velocity zones^39^. Understanding the composite effect of landscape sources and sinks on microplastic abundances in freshwater food webs requires comparisons across watersheds of different land-use types^40^.

The rapidly growing field of microplastic ecology has allowed for developments in methodology and thereby facilitated new approaches to data collection and processing^41–43^. Many studies of microplastic use neuston or plankton nets to collect samples^44^. However, Barrows *et al*.^42^ compared ‘grab’ samples of 1 L of seawater to the conventional neuston net approach and found grab samples collected 3 orders of magnitude (5.9 ± 4.4 SD L^−1^) more microplastic than the net approach (0.005 ± 0.004 SD L^−1^). The authors concluded most microplastic escaped the neuston net (0.363 mm mesh) and that grab samples collected a wider size range of microplastic, which better reflect microplastic abundance in the environment^42^.

In addition to changes in data collection procedures, contamination of samples with microplastic is common. Sources include airborne particles in the lab and field, as well as microplastic in reagent chemicals and filtered lab water^45,46^. Previous research accounts for contamination in each step^10^, but no previous studies have quantified the size, shape, and color of contamination particles compared to environmental samples. This comparison will help isolate the sources of contamination and more accurately account for contaminants in field samples.

In this study, we measured microplastic abundance in fish and water from three major tributaries of Lake Michigan, which spanned a land-use gradient. We expected *(H*_1_) that microplastic abundance in fish would increase with body size and trophic position, and *(H*_2_) that a positive relationship would be observed between microplastic concentrations in fish digestive tissue and the water column. In addition, we hypothesized *(H*_3_) that microplastic would predominantly be small or medium sized fibers across river sites and fish taxa, with a similar composition as a recent study using the same grab sample approach in the Gulf of Maine^43^. Finally, *(H*_4_) we predicted that analytical controls (to account for contamination) would show substantially fewer microplastic fibers and would be distinct in size, color, and relative composition compared to environmental samples.

## Methods

### Study Sites

**Figure 1:**
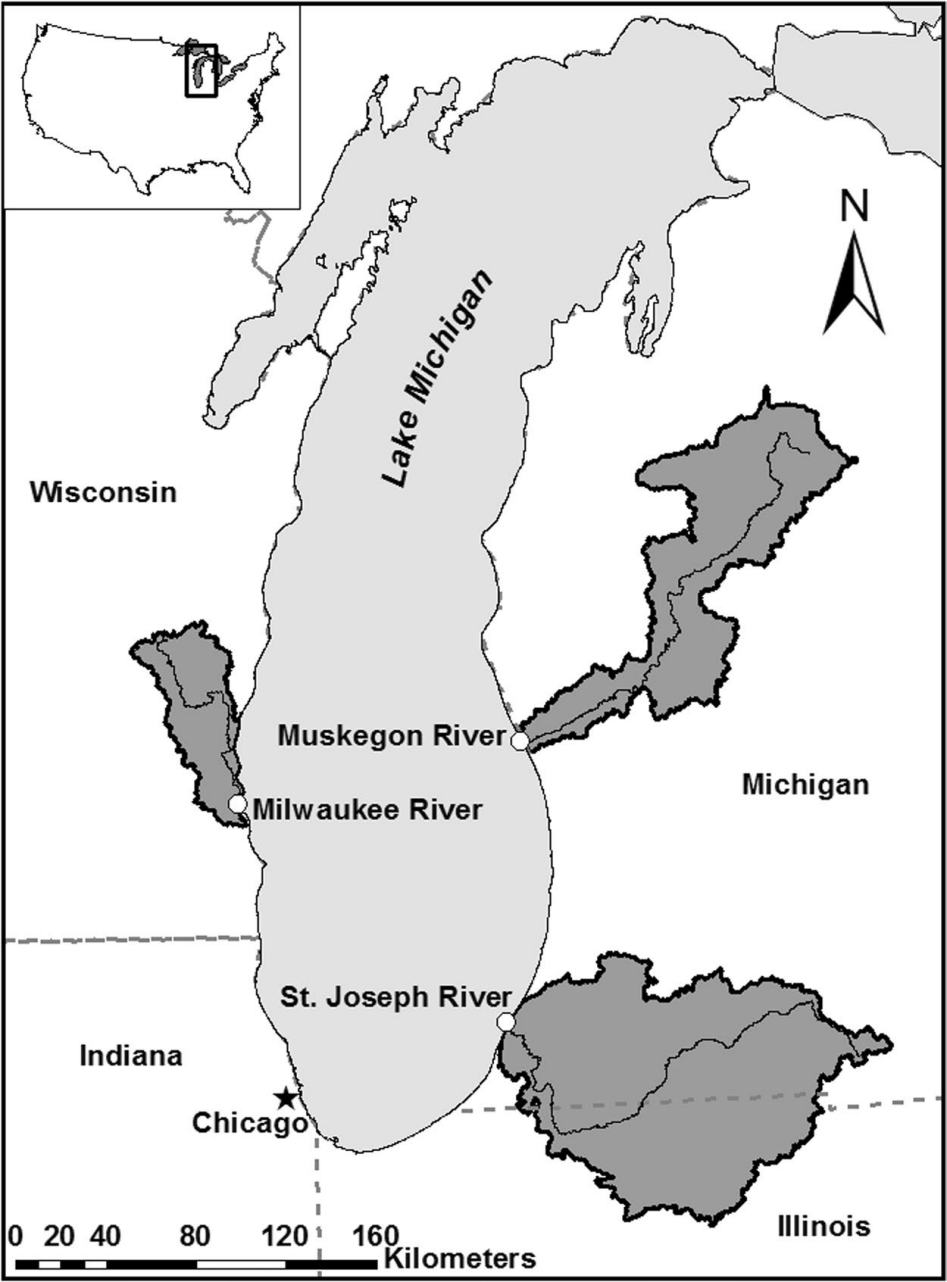
Muskegon River, Milwaukee River, and St. Joseph River watersheds and site locations around Lake Michigan, USA.

The Muskegon River (MG), Milwaukee River (MK), and St. Joseph River (SJ) are major tributaries of Lake Michigan, USA (Fig. 1). We selected these sites to span a land-use gradient dominated by forest (MG), urban-agriculture (MK), and agriculture (SJ; Table 1). Dominant land-use categories were determined by calculating relative abundance of land-use in forest, urban, and agriculture categories within each watershed using the 2011 National Land Cover Dataset in ArcMap Geographic Information Systems (GIS)^47^. All field work was conducted upriver of each river mouth to ensure surface water current was unidirectional toward Lake Michigan. The Muskegon River was sampled within Muskegon State Park, 1.1 km upstream from the river mouth in Muskegon, MI (43°13′56.2″N, 86°19′40.1″W). Milwaukee River field work was conducted 1.5 km upstream from the river mouth, just downstream of the confluence of the MK and Menomonee Rivers in Milwaukee, WI (43°01′49.8′N, 87°54′27.9″W). The St. Joseph River field site was 0.93 km upstream from the river mouth in Benton Harbor and St. Joseph, MI (42°06′44.4″N, 86°28′41.7″W). All field work was conducted with approval of state and local officials.

**Table 1:** Site description and watershed landscape features for three major tributaries of Lake Michigan.

Site	Dominant Land-Use	Forest (%)	Urban (%)	Agri. (%)	WWTP Discharges (No.)	Non-WWTP Discharges (No.)	Watershed Area (km^2^)	River Discharge (m^3^ s^−1^)
Muskegon	Forest	39.5	08.5	19.6	26	144	13,701	129.1
Milwaukee	Urban-Ag	12.1	30.3	42.7	14	106	1,803	7.8
St. Joseph	Agriculture	11.4	13.7	58.7	86	730	12,207	62.9

WWTP Discharges and Non-WWTP Discharges refer to wastewater treatment plants and other discharges, respectively, on the Environmental Protection Agency’s list of facilities that discharge wastewater to the rivers.

### Fish and Water Collection

Fish were opportunistically collected with wading seine nets adjacent to MG and SJ water collection sites and 1.5 km downstream from the MK water collection site. Fish were preliminarily identified in the field and up to 15 fish per taxa were euthanized with MS-222 (Tricaine-S; 0.25 g L^−1^) while the remaining fish were released. Fish were preserved in 70% ethanol in the field and transported to the laboratory where they were identified to species or genus^48^ and processed for microplastic gut content. All methods were carried out in accordance with ethical guidelines and regulations and approved by Loyola University Chicago’s Institutional Animal Care and Use Committee.

Microplastic was collected from surface water habitats via grab samples with 2 L glass bottles (*n* = 4 bottles per site) from the MG, MK, and SJ Rivers during summer 2016. Bottles were rinsed three times with DI water filtered through a 0.363 µm mesh in the lab pre-sampling. Collection bottles were filled with water directly below the river surface along the left and right side of river channels (*n* = 2 bottles per in-stream river location), capped immediately to prevent atmospheric contamination of microplastic, and transported to the laboratory for microplastic processing^42^.

### Microplastic Quantification and Characterization

In the laboratory, the body length of each fish was measured, the digestive tracts were dissected, and the tissue was placed in individual clean beakers. Digestive tissue was dried at 75 °C for at least 24 h and underwent wet peroxide oxidation (0.05 M Fe(II) and 30% H_2_O_2_) at 75 °C to dissolve organic material^49^. Microplastic is resistant to wet peroxide oxidation^49^. Samples were filtered onto gridded 0.45 µm filters (Whatman^TM^, Pittsburgh, Pennsylvania, USA). Filters were examined at 25–50 × magnification under dissecting microscopes and checked two separate times to confirm microplastic counts were consistent and conservative. Microplastic was counted and categorized as either fiber, fragment, bead, foam, or film and classified into a color category^20,50^. Due to the high abundance of fibers, length was measured on a randomly selected sub-sample of fibers on each filter. Fiber length was measured along the longest dimension with an ocular micrometer or estimated using the filter grid width (3.2 mm)^42^. We measured size and color of 554 fibers from fish (*n* = 819 counted). Fish were classified into functional feeding groups (FFG) and assigned a trophic fraction based on collective data available on FishBase^51^ coupled with visual identification of macroinvertebrates in gut content post wet peroxide oxidation (Table 2). A total of 17 fish were classified as detritivores, 30 as omnivores, and 27 as zoobenthivores (Table 2). Microplastic concentration in fish taxa and FFG were calculated as the No. microplastic fish^−1^.

**Table 2:** Fish functional feeding group classification, trophic position, and abundance collected from the Muskegon, Milwaukee, and St. Joseph Rivers.

Taxa	Common Name	Functional Feeding Group	Trophic Fraction	Abundance	Size Range (cm)	No. of Fish with Microplastic
*Dorosoma cepedianum*	Gizzard Shad	Detritivore	2.40	1	7.6	0
*Catostomus commersonii*	White Sucker	Detritivore	2.46	16	4.5–12	15
*Pimephales promelas*	Fathead Minnow	Omnivore	2.80	10	5.6–6.5	6
*Carpoides cyprinus*	Quillback	Omnivore	2.59	1	9.0	1
*Notropis stramineus*	Sand Shiner	Omnivore	2.37	17	3.9–6.9	16
*Notropis hudsonius*	Spottail Shiner	Omnivore	2.74	2	5.5–5.6	2
*Fundulus diaphanus*	Banded Killifish	Zoobenthivore	3.18	4	4.5–6.7	4
*Micropterus sp*.	Bass sp.	Zoobenthivore	4.09	3	5.6–7.3	3
*Notropis atherinoides*	Emerald Shiner	Zoobenthivore	2.80	2	6.8–8.4	2
*Neogobius melanostomus*	Round Goby	Zoobenthivore	3.30	14	5.1–9	14
*Cyprinella spiloptera*	Spotfin Shiner	Zoobenthivore	3.24	4	5.0–8.1	4

River surface water samples were vacuum filtered onto gridded 0.45 µm filters (~300 mL filter^−1^) and oven dried at 75 °C for 24 h^42^. Microplastic was quantified as explained above. Microplastic concentration was calculated by dividing the total number of microplastic in each sample by the sample’s total volume (L).

Microplastic polymer type was identified using Fourier Transform Infrared Spectroscopy (FTIR) on randomly selected microplastic from environmental samples. This technique produces infrared absorption bands that are unique to each polymer type. The small size of most of the microplastic and impurities (*e.g*., organic material and minerals) that can adhere to the microplastic is challenging for FTIR^14,52,53^. Of 160 number of fibers analyzed by FTIR, 5.6% were successfully identified.

### Quantification of Laboratory Microplastic Contamination

We carried out filter controls to account for microplastic contamination associated with surface water samples. We placed gridded 0.45 µm filters onto the vacuum filtration apparatus and rinsed the collection cup with 0.363 µm filtered DI water (*n* = 10 filter controls). In addition, a second set of controls were completed to account for microplastic contamination from the digestion and filtering processes (*i.e*., ‘digestion control’) that fish samples were exposed to. Twenty mL of 30% H_2_O_2_ and 20 mL of 0.05 M Fe(II) solution were added to a clean beaker, heated at 75 °C, and vacuum filtered onto a gridded 0.45 µm filter (*n* = 10). Microplastic was quantified and characterized as explained for surface water and fish samples. Microplastic contamination (mean No. filter^−1^) consisted of 2.3 (±0.63 SE) and 4 (±0.39 SE) fibers for filter and digestion controls, respectively. Mean fiber contamination from each control was accounted for in each sample type (*i.e*., digestion control was used for fish and filter control was used for grab samples). We corrected fiber color and size category by removing fibers from fish (4) and surface water samples (2) following the color and size class frequencies recorded on control filters. Finally, we compared the abundance, category, size, and color of contamination fibers to those found in environmental samples.

### Statistical Analyses

We compared microplastic concentration in fish among sites and taxa using both one-way ANOVA and Kruskal-Wallis analyses with *anova(lm())* and *kruskal.test()* using the R statistical program^54^. We used the same test to compare surface water microplastic concentrations among sites. Presentation of both non-parametric and parametric analyses were included to balance the interpretation of the lower power non-parametric test with the robust parametric analyses since data sets were a mix of both normal and non-normal Gaussian distributions, which were similar to analyses conducted with our previous work^55^. To identify which sites were significantly different from one another, pairwise post-tests were conducted between sites with both *pairwise.t.test()* with a Bonferroni correction (parametric) and *post.hoc.kruskal.nemenyi.test()* (non-parametric) in ‘PMCMR’ R^56^. Microplastic concentration categorized by fish FFG (*i.e*., detritivore, omnivore, zoobenthivore) was non-normal and analyzed with the Kruskal-Wallis test^54^ followed by *post.hoc.kruskal.nemenyi.test()* to determine which FFG was significantly different from one another.

We conducted linear regression analyses between fish microplastic concentration and surface water microplastic concentration using *lm()*. We also conducted linear regression analyses between fish traits (*i.e*., body size and trophic fraction) and microplastic concentrations in fish with linear regression models to determine if microplastic abundance in fish increased with increasing fish body size and trophic fraction. To determine if larger fish ingested larger fibers, fiber length and fish body length were also analyzed with linear regression models. All fish trait regression models were conducted with pooled and individual species data, with the exception of fish trophic fraction (*i.e*., each taxa was assigned one trophic fraction).

Microplastic category, size class, and color patterns were analyzed with Chi-square test of independence after data were converted to ratios for each sampling site with *chisq.test()*. This analysis also included data collected from the Gulf of Maine using the same grab sampling and laboratory processing methods as used in our study^42^. These analyses allowed for a test of independence to determine if microplastic category, size class, or color patterns were independent of sampling sites^57^. To identify if microplastic patterns in the environment were similar to patterns found in laboratory controls, Chi-square test of independence analyses were conducted with microplastic category, size class, and color data (surface water and fish) and their respective controls (*i.e*., filter and digestion). Microplastic concentration comparisons between environmental data and controls were analyzed with student’s t-test (*t.test()*) and Wilcoxon rank-sum test, (*wilcox.test()*), after data were found to have mixed normality post Shapiro-Wilk normality tests^54^. All analyses were conducted using the ‘base’ package in R version 3.3.0 unless stated otherwise.

### Data Availability

Data will be made available when requested from corresponding author.

### Ethical Statement

All methods were carried out in accordance with ethical guidelines and regulations and approved by Loyola University Chicago’s Institutional Animal Care and Use Committee.

## Results

### Microplastic Abundance

**Figure 2:**
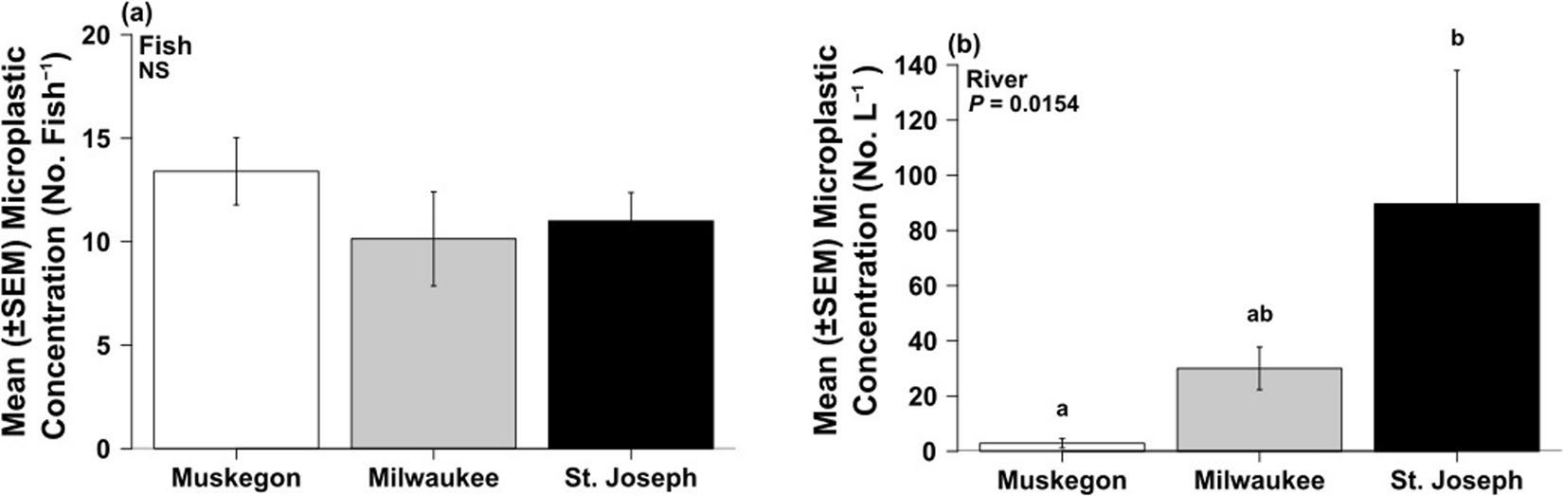
Mean microplastic concentration in fish (**a**) and mean microplastic surface water concentration (**b**) between Muskegon, Milwaukee, and St. Joseph Rivers during summer 2016. Letters indicate significant difference between sites at *P* < 0.05.

**Figure 3:**
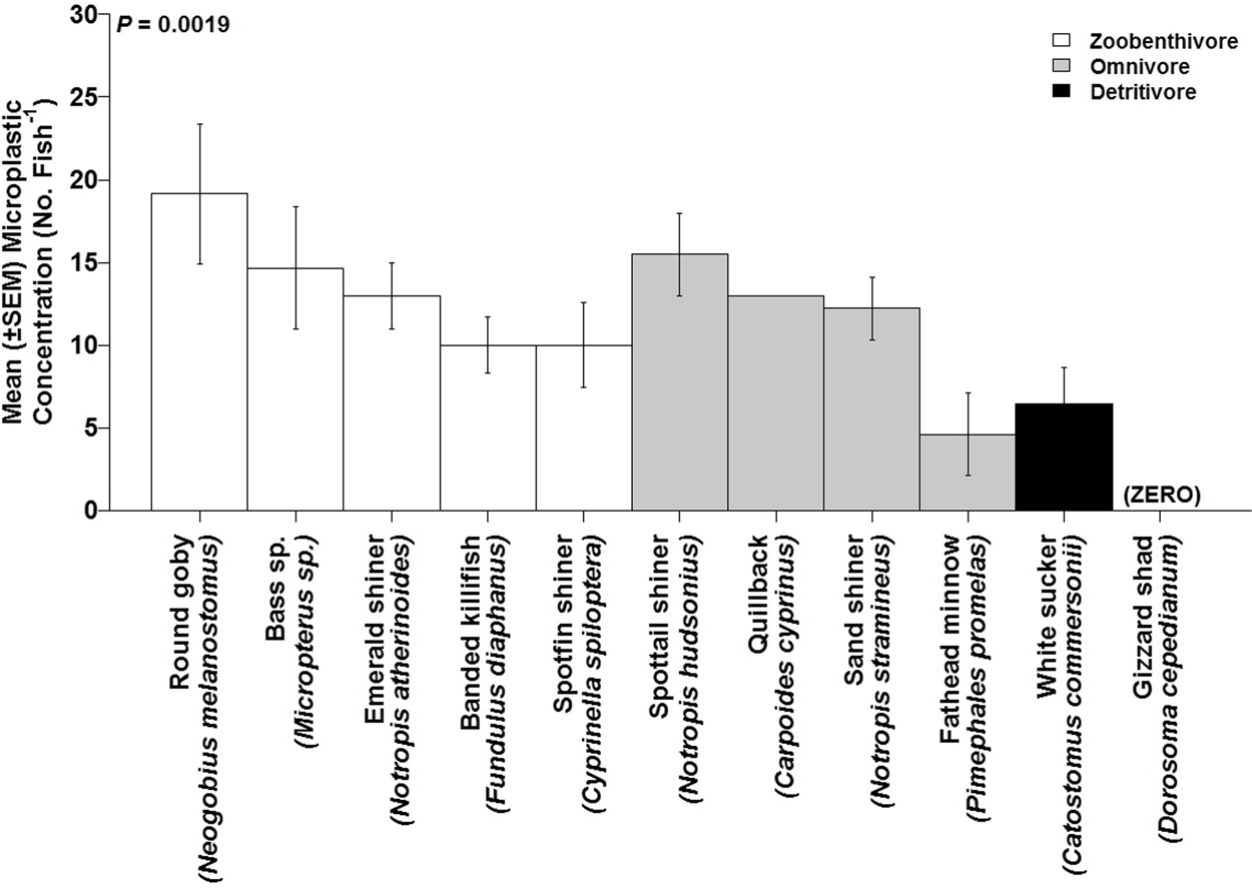
Mean microplastic between fish taxa categorized as either zoobenthivore, omnivore, or detritivore. Zero indicates there was no microplastic present in the indicated taxa.

A total of 74 fish spanning eleven taxa were collected throughout the study, with 10 taxa (85% of individuals) containing microplastic in their digestive tracts (Table 3). Microplastic abundance in fish was not significantly different among the three study sites (*P* > 0.05) and ranged from 10 (±2.3) to 13 (±1.6) microplastic particles fish^−1^ (Table 3; Fig. 2a). However, there was a significant difference in microplastic concentration across fish species (Table 3; Fig. 3). Round goby (*Neogobius melanostomus*) microplastic concentration was significantly greater than fathead minnow (*Pimephales promelas*) and white sucker (*catostomus commersonii*) (all *P* < 0.05), with no microplastic in gizzard shad (*Dorosoma cepedianum*) (Fig. 3). Round goby was the only fish present across all three sites, and gobies from SJ River had approx. 50% less microplastic concentration compared to those collected from the MG and MK Rivers (Suppl. Table 1). Microplastic concentrations in surface waters were significantly different among the 3 sites (Fig. 2b, Table 3), and abundance in fish was unrelated to patterns in the water (*r*^2^ = −0.011, *P* > 0.05).

**Table 3:** One-way ANOVA and Kruskal-Wallis statistical analyses for mean microplastic concentration, flux, and export between Muskegon, Milwaukee, and St. Joseph Rivers, and microplastic within fish between sites, taxa, and functional feeding groups during summer 2016.

Sample Type	df	ANOVA		Kruskal-Wallis	
		F-Value	*P*-Value	*X* ^2^	*P*-Value
Concentration	2	2.46	0.140	08.35	0.015
Fish Sites	2	0.50	0.609	08.14	0.017
Fish Taxa	10	1.95	0.054	27.83	0.002
Fish FFG	2	—	—	14.18	0.001

There was also a significant effect of fish FFG on microplastic concentration in fish regardless of river site (Table 3), with zoobenthivores greater in microplastic concentration than detritivores (*P* < 0.001). Zoobenthivore fish had significantly greater microplastic compared to the omnivore FFG in the MK River (*X*^2^ = 10.92, df = 2, *P* < 0.01), whereas there was no difference in microplastic abundance among FFG present in other river sites (all *P* > 0.05; Suppl. Fig. 1). Omnivore fish concentration was significantly influenced by site (F = 4.92, df = 2, *P* = 0.015), with omnivore fish from the MG River significantly greater in microplastic concentration compared to omnivore fish from the MK River (*P* = 0.018) but was similar in concentration to fish from the SJ River (*P* > 0.05). Zoobenthivore fish in the MK River had the greatest microplastic concentration compared to zoobenthivore fish from MG and SJ Rivers; however, this comparison was not statistically significant (F = 2.169, df = 2, *P* > 0.05). Fiber polymer composition from fish digestive tissues consisted of polyethylene, polyacrylonitrile, polyacetal, and polyvinyl acetate (Suppl. Table 2).

**Figure 4:**
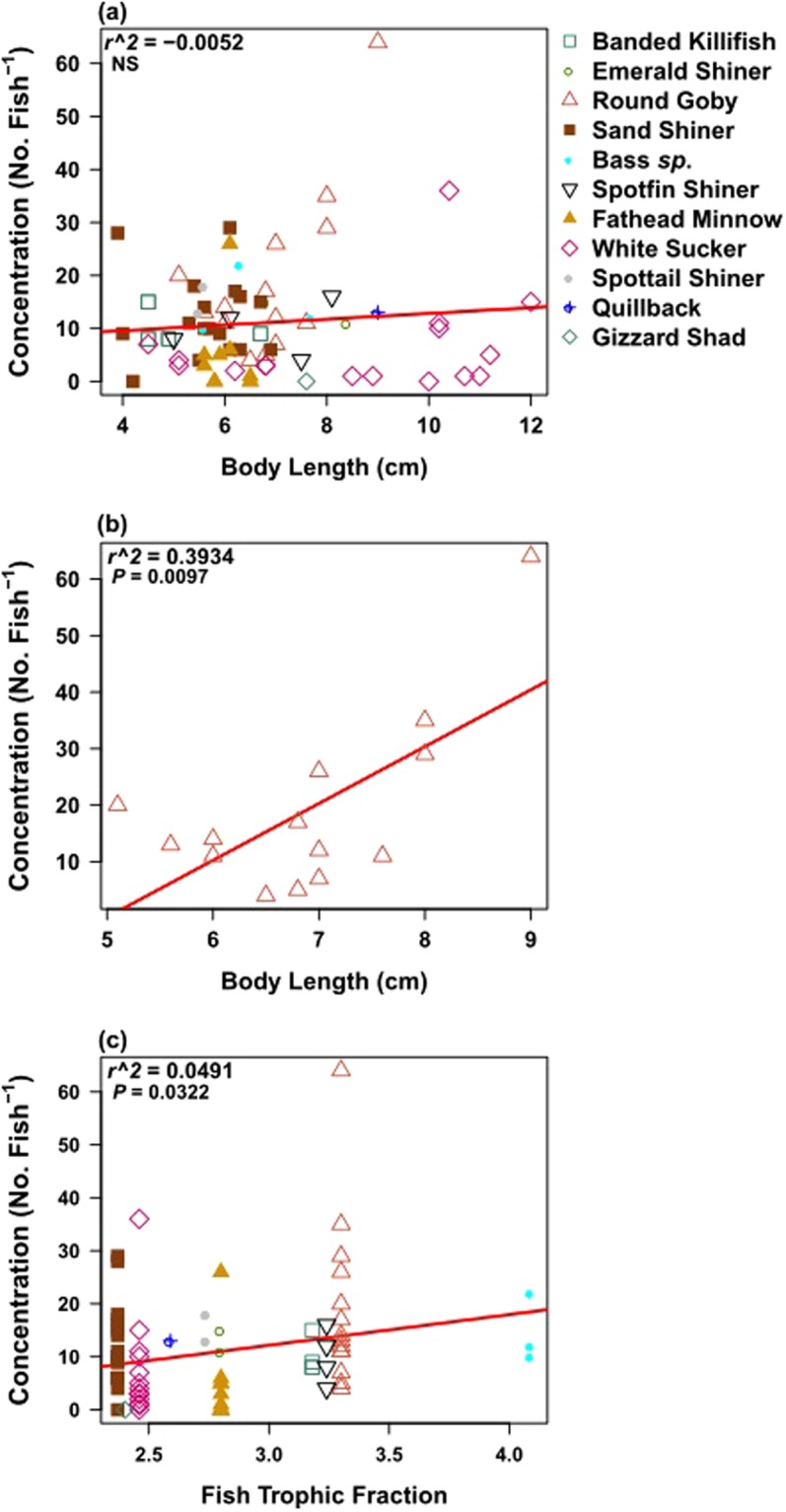
Linear regression analysis for number of microplastic within pooled fish and fish body size (**a**) round goby size and number of microplastic (**b**), and fish trophic fraction and number of microplastic (**c**).

There were some links between fish body size and trophic fraction and the abundance of microplastic in digestive tissue. For example, gut microplastic in round goby increased with body length (*r*^2^ = 0.393; *P* = 0.010; Suppl. Table 3; Fig. 4b), but fish body size was not related to microplastic abundance in other taxa or when taxa were pooled (Suppl. Table 3; Fig. 4a). Microplastic abundance in fish was also positively related to an increase in fish trophic fraction, although the model explained a small portion of the variation in the data (*r*^2^ = 0.050; *P* = 0.032; Fig. 4c). Microplastic abundance in zoobenthivores was positively related to increasing fish body length (*r*^2^ = 0.188; *P* = 0.014; Suppl. Table 3), which was most likely driven by the round goby (classified as zoobenthivore; Table 2). There were no such patterns observed for the other FFG (Suppl. Table 3). Fiber length within fish digestive tracts had no relationship with fish body length, indicating fibers of all size classes were found equally in small and large fish (Suppl. Table 4).

### Characterization of Microplastic

**Figure 5:**
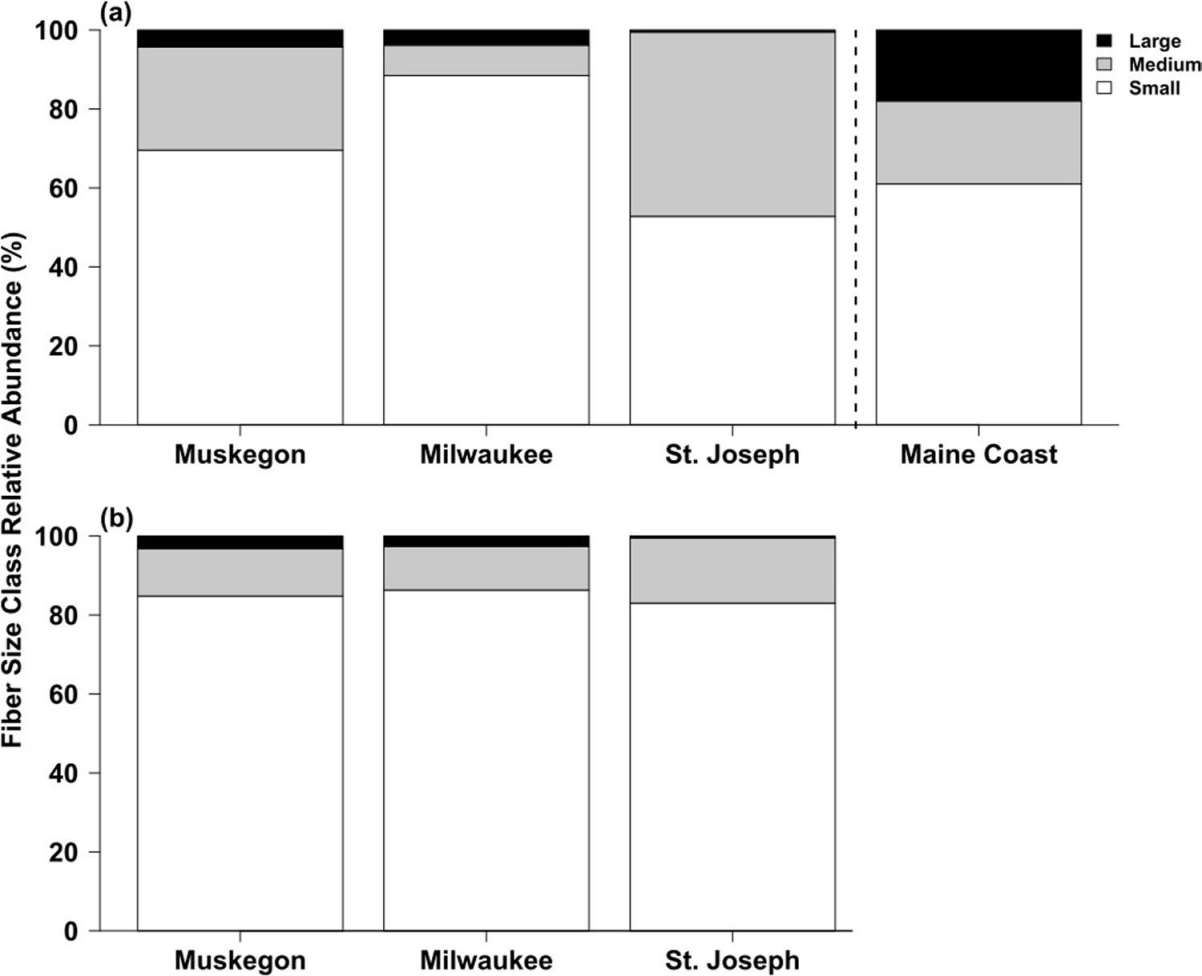
Relative abundance of fiber size in surface water (**a**) and fish (**b**) collected from the Muskegon, Milwaukee, and St. Joseph Rivers. Maine coast refers to surface water fiber size from Barrows *et al*.^42^ collected off the coast of Maine USA.

Microplastic category, size class, and color patterns were significantly different in surface water river sites and the Gulf of Maine^42^ (Fig. 5, Suppl. Table 5, Suppl. Figs 2 and 3). Fibers comprised approximately 97–100% of all microplastic found in the water and fish (Suppl. Fig. 2). Fragments comprised 1.5–3% of microplastic collected from water in the MK and SJ Rivers respectively. Foam was only found in the MK River and accounted for 0.4% of the water column microplastic at that site. Fragments were also rare in fish and accounted for approximately 2.5–3% of the microplastic (Suppl. Fig. 2). The relative abundance of fiber colors significantly depended on river site (*X*^2^ = 66.06, df = 6, *P* < 0.001), with clear fibers dominant at the SJ River (approx. 80%) and blue fibers (approx. 44%) most common at the MG River (Suppl. Fig. 3a). In contrast, fiber color patterns were similar in fish across sites, with clear and blue fibers predominant (Suppl. Fig. 3b). A total of 526 (out of 980) fibers were classified as small (<1.5 mm), medium (1.6–3.2 mm), or large (>3.3 mm). Small fibers were the most common size found across water samples and fish (Fig. 5). However, medium sized fibers were common from water samples at the SJ River but not at the other sites (Fig. 5a). Surface water microplastic category, color, and fiber size class patterns in the Gulf of Maine were similar to data from the MG, MK, and SJ Rivers (Fig. 5a; Suppl. Figs 2a and 3a).

### Characterizing Microplastic in Laboratory Controls

**Figure 6:**
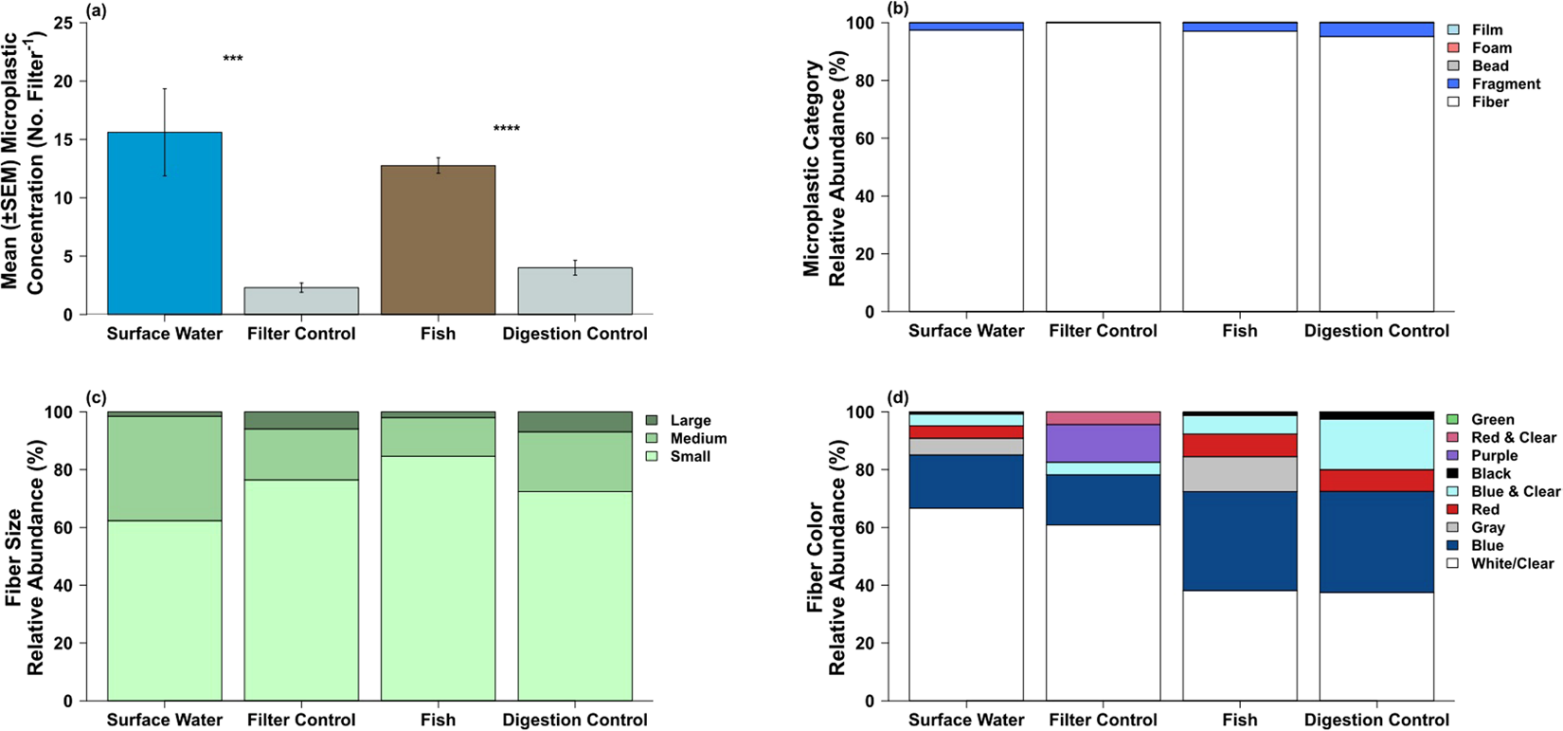
Surface water and fish samples compared to their corresponding lab controls for microplastic concentration (**a**), category (**b**), fiber size (**c**), and fiber color (**d**). An asterisk indicates significant difference at ^***^P < 0.01 and ^****^P < 0.001.

Analyses of microplastic in control procedures could help reveal potential sources of microplastic contamination. Microplastic concentrations were significantly lower in laboratory controls compared to environmental samples (Fig. 5a; t_River_ = −3.544, df = 72.5, *P* < 0.001; t_Fish_ = 9.563, df = 37.4, *P* < 0.001). Microplastic concentrations on surface water (approx. 16 ± 4 pieces filter^−1^) and fish filters (approx. 13 ± 0.7 pieces filter^−1^) were 8× and 3× greater than filter controls (2 ± 0.4 pieces filter^−1^) and digestion controls (4 ± 0.63 pieces filter^−1^; Fig. 6a) respectively. Microplastic composition in filter and digestion controls was different in size class and color patterns compared to water column and fish results (Suppl. Table 6; Fig. 6b,c). Fibers were the dominant microplastic type found in controls, which was similar to surface water and fish (Fig. 6b). Small fibers were the dominant size class found across environmental samples and controls. However, large fibers were more common in filter controls than surface water samples (Fig. 6c). Clear was the dominant color in both surface water and filter control samples (Fig. 6d), but purple and red/clear bi-colored fibers were more common in filter controls compared to surface water samples. Similarly, red and gray fibers were common in the water samples, but not in filter controls (Fig. 6d). Clear and blue fibers were the most common fibers in both fish and digestion controls, but digestion controls were characterized by more blue/clear fibers compared to fibers from fish, which had more gray fibers (Fig. 6d).

## Discussion

Microplastic pollution is pervasive worldwide, but research on its abundance, movement, and biological interactions in freshwater ecosystems is newly emerging. In this study, we present evidence that microplastic is present in major tributaries of Lake Michigan, including fish digestive tissue and surface waters. Understanding the factors which drive microplastic patterns in freshwater food webs will be critical for management policies.

### Microplastic in fish digestive tracts was different among species and feeding groups

Plastic and other anthropogenic litter is commonly found in marine fish^58^, but its abundance, introduction pathway, and physiological effects on freshwater fish are largely unknown. In this study, 85% of fish had microplastic in their digestive tissues with an average of approximately 13 particles fish^−1^ with fibers the dominant microplastic. Pazos *et al*.^59^ found microplastic abundance was approximately 8–55 particles fish^−1^, and fibers were also the dominant microplastic category across all fish taxa and from fish collected from the Rio de la Plata estuary, Argentina^59^, which is comparable to gut microplastic in fish in our study. In contrast, Lusher *et al*.^60^ found 11% (84 of 761) of mesopelagic fish (*e.g*., glacial lantern fish (*Benthoseoma glacial*)), spotted barracudina (*Arctozenus risso*), and lancet fish (*Notoscopelus kroyeri*) from the Northeast Atlantic Ocean had microplastic, with an average of 1.2 particles fish^−1^. Anthropogenic litter has been found in 25–28% of fish collected from markets prepared for human consumption (seafood) in the USA and Indonesia ranging from 0.3–5.9 particles fish^−1 ^^58^. These studies suggest microplastic abundance in fish could vary across a gradient of aquatic habitats. Microplastic in riverine fish might be higher than marine fish due to lower water volume:surface water area ratio and proximity to terrestrial microplastic point-sources; however, a systematic comparison has not yet been completed.

Fish ecological and morphological traits were linked to gut microplastic abundance from fish in Lake Michigan major tributaries. As we expected, microplastic abundance was positively related to fish trophic fraction. Zoobenthivores had greater microplastic abundance compared to omnivores and detritivores, suggesting predator oriented fish may obtain microplastic via trophic transfer from prey items. Farrell *et al*.^24^ demonstrated the potential for microplastic trophic transfer from mussels (*Mytilus edulis*) to crabs (*Carcinus maenas*). The authors reported microplastic concentrations were up to 163,111 particles mL^−1^ of crab haemolymph, which represented 0.24% of the microplastic retained by mussels. Two fish species of particular interest in our study were the round goby and the gizzard shad. The round goby is invasive in the Great Lakes and is a voracious, opportunistic feeder^61,62^. This could explain why this species had the highest microplastic concentration in its digestive tissues (approx. 20 particles fish^−1^). Microplastic abundance in round gobies increased with fish body length and suggested microplastic in these fish could accumulate with age. The gizzard shad was the only taxa with no microplastic in its gastrointestinal tract, which could be attributed to its common diet of plants and detritus instead of aquatic fauna^63^. In contrast, Pazos *et al*.^59^ found no relationship between microplastic abundance in fish and fish trophic group and body length in the Rio de la Plata estuary, Argentina. Ferreira *et al*.^64^ reported 64% of *Cynoscion acoupa* (Acoupa weakfish, Lacepéde) collected from the Goiana Estuary (South America) had microplastic in their stomachs, with adult fish containing more microplastic than juvenile and sub-adult fish, suggesting fish ontogeny may play a role in microplastic abundance in fish. Collective findings from this study suggest fish species traits could help explain microplastic abundance, but can be species dependent. Future research should include identifying functional traits linked with microplastic abundance in aquatic biota to help identify wildlife taxa susceptible to microplastic.

These results do not indicate the source(s) of microplastic in fish digestive tracts. Microplastic abundance in fish were similar across the 3 study sites, indicating microplastic concentration in the water column was not a reliable predictor of microplastic abundance in fish and may not be the primary source of ingested microplastic for these taxa. In contrast, Pazos *et al*.^59^ found fish collected closer to WWTPs had significantly greater microplastic concentrations in their digestive tissues compared to fish from further locations in the freshwater zone of the Rio de la Plata estuary, Argentina. Most studies have focused on quantifying microplastic abundance in fish and invertebrates^65,66^, but little research has been conducted to identify the movement of microplastic between the environment and organisms^24^. Aquatic biota may ingest microplastic either directly from their habitat (*i.e*., water column or benthos) or indirectly via trophic transfer. It is unknown the proportion of microplastic each pathway contributes to microplastic abundance in aquatic biota, or what aspects of gut tissue anatomy may affect internal microplastic retention. Retention of microplastic in the gut could lead to irritation of the epithelial lining and blockage in the digestive tract due to the shape and filamentous structure of microplastic^25^, which could impact aquatic biota ingestion and egestion rates. Future research is needed to explore the pathways in which microplastic is incorporated into aquatic biota and food webs.

### Sources of microplastic to rivers

Watersheds with urban and agricultural land-use can have increased point- and non-point sources of microplastic pollution. We noted higher microplastic concentration was in human-dominated watersheds relative to the forested watershed. Some microplastic sources in urban and agricultural environments are well documented, while others will require additional research to measure. For example, McCormick *et al*. (2016) reported microplastic flux downstream of WWTPs in the Chicago region, a densely populated area in the USA, was on average 1.3 million particles d^−1^ and was higher downstream of WWTP outfalls compared to upstream locations. Litter can accumulate on freshwater beaches and in riparian zones of rivers^27,67^ and is similar in composition in benthic habitats^28^, suggesting urban terrestrial zones are sources of AL, and possibly microplastic, to rivers. Plasticulture, the use of plastic film/mulch to cover and protect seedbeds, is a worldwide agricultural practice that could also contribute microplastic to the landscape across large spatial scales. For example, plasticulture is present in 156,900 ha in the Shandong province, China^68^, creating the potential for microplastic to be introduced throughout this region as large plastic degrades into smaller particles. Microplastic concentrations in biosolids (*i.e*., WWTP sludge converted to a fertilizer) from seven WWTPs in Ireland ranged from 4,196–15,385 particles kg^−1^ dry weight^36^. Therefore, biosolid application on agricultural fields may be a non-point source of microplastic pollution to nearby rivers and lakes; however, this has not yet been measured.

### Comparing results to literature and considerations for scaling

Comparing the values for water column microplastic from this study to published results requires consideration of methodology and location. Previous work using neuston nets in freshwater ecosystems found lower values than those documented with grab samples. In a study measuring microplastic abundance in 29 Laurention Great Lakes tributaries using neuston nets, microplastic concentrations ranged from 0.05–32 particles m^−3^ and was positively related to an increase in urban land-use^40^. The Seine River at Paris had microplastic concentrations of 3–106 particles m^−3 ^^11^ with the same method. Finally, McCormick *et al*. (2016) also used neuston nets to report microplastic concentrations downstream from WWTP effluent (0.80–11.22 particles m^−3^) were greater than concentrations upstream of WWTPs (0.48–5.92 particles m^−3^). In contrast, a grab sample approach in the Gulf of Maine estimated microplastic concentrations at 3,400–10,000 particles m^−3 ^^42^, approximately one order of magnitude less than our results. Barrows *et al*.^42^ also reported similar composition of microplastic as our results, where particles were primarily small fibers (<1.5 mm) and most commonly clear or blue^42^ (supporting *H*_4_). These collective results show microplastic pollution is abundant in freshwater habitats and that rivers are sources of microplastic pollution to downstream ecosystems.

Scaling up microplastic results to larger volumes of water and time periods will require careful attention to replication and consideration of distinct river habitats. We did not extrapolate our water column samples to estimate flux (No. particles day^−1^) or export (No. particles km^−2^ d^−1^) due to the volume and lack of temporal replication in our surface water collection. Estimates of flux and export are needed to inform global budgets of plastic movement in rivers. To do so, we suggest that more samples should be collected across the width of a river, with depth-integrated collection, and at multiple times of year. In addition, we propose that investigators simultaneously measure microplastic at the benthic surface, in the water column, and floating at the water surface. Initial assessments of these habitats in rivers suggest high variation among sampling locations and times, including deposition and resuspension of microplastic, as is common for naturally occurring particles^39^. Rigorous sampling regimes will allow for initial budgets of microplastic to be constructed for rivers.

### Composition of microplastic in controls

Analyses of microplastic in control procedures could help reveal potential sources of microplastic contamination in laboratory settings. Laboratory microplastic contamination was minimal in this study (2–4 particles filter^−1^) and similar to other studies^20,59^ (*e.g*., McCormick *et al*.^20^, Pazos *et al*.^59^). As we predicted, microplastic in controls were typically large fibers and unique in colors (*e.g*., purple) compared to environmental samples. Sources of microplastic contamination could have come from laboratory technician clothing, atmospheric deposition, and the water supply. In this study, DI water with a 363 µm mesh covering the faucet was used for all solutions and rinsing of glassware. De-ionized water was used due to greater microplastic contamination in MilliQ and tap water in the laboratory (*personal observation*, McNeish). Microplastic contamination has been documented in a diversity of commercial salt brands ranging from 1–10 particles kg^−1^ of salt^46^, which is important since density separation of microplastic using salts is a common protocol in the microplastic field^49^. Possible contamination may have also come from pre-ordered 30% hydrogen peroxide. Although we did not isolate the sources of microplastic contamination, this study is the first to report microplastic contamination is unique in size and color compared to environmental samples.

Polymer identification in small plastic fibers is a major challenge for this field of research. It is possible some fibers in this study were mis-identified as plastic instead of other anthropogenic sources of fibers (*e.g*., cotton and viscose fibers). Lenz *et al*.^69^ reported a 25% error in mis-identification of fibers as plastic when comparing visual versus FTIR identification of fibers as plastic. This suggests the possibility that 25% of the fibers in this study could not be plastic. However, the fibers that were identified by FTIR in our study were all plastic. Moreover, the patterns observed across sites and fish taxa would still be the same assuming this error was consistent throughout sample processing. As technology for polymer identification on small fibers develops, this outstanding question of misidentification will inform research on microplastic pollution across ecosystem types.

### Summary

Microplastic is abundant in rivers and fish connected to Lake Michigan, which serve as conduits of contamination via river currents and fish movement. Species functional traits may help predict microplastic abundance in fish and could be applied to other fauna. Understanding traits that make fauna susceptible to microplastic pollution could enhance our understanding of how these organisms interact with microplastic and could enable us to target species for conservation efforts. Microplastic concentrations in Lake Michigan tributaries were also higher than what has been reported for marine coastal habitats^42^ and the open ocean^70^; although, greater harmony in methodological approaches would be needed for more robust comparisons of microplastic concentrations across large spatial scales. In particular, more research is needed to pinpoint the landscape features which serve as point and non-point sources of microplastic pollution to freshwaters, and its accumulation in food webs. These collective findings highlight the need for future research to focus on the movement of microplastic across the terrestrial-aquatic boundary and the importance of focusing pollution management efforts on inland waters.

## Electronic supplementary material

Supplemental Materials
