# Supplementary material for: Microplastic in riverine fish is connected to species traits
Source: Sci Rep. 2018 Aug 3;8:11639. doi: 10.1038/s41598-018-29980-9 (PMC6076259; doi:10.1038/s41598-018-29980-9)

**Supplemental Materials**

**Title:** Microplastic in riverine fish is connected to species traits

**Authors:** RE McNeish, LH Kim, HA Barrett, SA Mason, JJ Kelly, TJ Hoellein

Supplemental Table 1: Mean microplastic fiber, fragment, bead, foam, film, and total concentration for each fish taxa collected from the Muskegon, Milwaukee, and St. Joseph Rivers. ***^†^***Indicates fish species was present across all river sites.

| **Site** | **Taxa** | **Fiber** | **Fragment** | **Bead** | **Foam** | **Film** | **Total** |
| --- | --- | --- | --- | --- | --- | --- | --- |
| Muskegon | *Cyprinella spiloptera* | 10.0 | 0.0 | 0.0 | 0.0 | 0.0 | 10.0 |
|  | *Fundulus diaphanus* | 9.8 | 0.3 | 0.0 | 0.0 | 0.0 | 10.0 |
|  | *Micropterus dolomieu* | 12.0 | 0.0 | 0.0 | 0.0 | 0.0 | 12.0 |
|  | *Neogobius melanostomus****^†^*** | 20.0 | 0.0 | 0.0 | 0.0 | 0.0 | 20.0 |
|  | *Notropis atherinoides* | 13.0 | 0.0 | 0.0 | 0.0 | 0.0 | 13.0 |
|  | *Notropis stramineus* | 15.8 | 0.8 | 0.0 | 0.0 | 0.0 | 16.6 |
| Milwaukee | *Catostomus commersonii* | 5.9 | 0.5 | 0.0 | 0.0 | 0.0 | 6.4 |
|  | *Micropterus sp.* | 10.0 | 0.0 | 0.0 | 0.0 | 0.0 | 10.0 |
|  | *Neogobius melanostomus****^†^*** | 22.6 | 0.3 | 0.0 | 0.0 | 0.0 | 22.9 |
|  | *Pimephales promelas* | 4.6 | 0.0 | 0.0 | 0.0 | 0.0 | 4.6 |
| St. Joseph | *Carpoides cyprinus* | 10.0 | 3.0 | 0.0 | 0.0 | 0.0 | 13.0 |
|  | *Cyprinella spiloptera* | 10.0 | 0.0 | 0.0 | 0.0 | 0.0 | 10.0 |
|  | *Dorosoma cepedianum* | 0.0 | 0.0 | 0.0 | 0.0 | 0.0 | 0.0 |
|  | *Micropterus dolomieu* | 22.0 | 0.0 | 0.0 | 0.0 | 0.0 | 22.0 |
|  | *Neogobius melanostomus****^†^*** | 10.5 | 0.0 | 0.0 | 0.0 | 0.0 | 10.5 |
|  | *Notropis hudsonius* | 15.5 | 0.0 | 0.0 | 0.0 | 0.0 | 15.5 |
|  | *Notropis stramineus* | 10.0 | 0.4 | 0.0 | 0.0 | 0.0 | 10.4 |

Supplemental Table 2: Summery of microplastic fiber polymer identification with FTIR.

| **Site** | **Sample Type** | **Color** | **Polymer** |
| --- | --- | --- | --- |
| Milwaukee | Fish | Blue | Polyethylene (PE) |
| Milwaukee | Fish | Clear | Polyacrylonitrile |
| Milwaukee | Fish | Clear | Polyethylene (PE) |
| Milwaukee | Fish | Blue | Polyacetal |
| Muskegon | Fish | White | Polyacetal |
| Muskegon | Fish | Black | Polyvinyl Acetate |
| Muskegon | Fish | Blue | Polyacrylonitrile |
| Muskegon | Fish | Clear | Polyethylene Terephthalate (PETE) |
| St. Joseph River | Bulk | Clear | Polyacrylonitrile |

Supplemental Table 3: Linear regression analyses for number of microplastic in fish taxa and FFG related to body length from the Muskegon, Milwaukee, and St. Joseph River watersheds. Analyses were conducted on fish taxa with *n* ≥ 3.

| **Taxa** | **Coeff.** | **Stand. Error** | **t-value** | ***P*-Value** | **Mult. R^2^** | **Adj. R^2^** | **F-Statistic** | **df** |
| --- | --- | --- | --- | --- | --- | --- | --- | --- |
| Pooled | 00.543 | 0.960 | 0.787 | 0.434 | 0.009 | -0.005 | 0.620 | 1,72 |
| Banded Killifish | -00.906 | 2.174 | -0.417 | 0.717 | 0.080 | -0.380 | 0.174 | 1,2 |
| Bass sp. | -00.204 | 6.009 | -0.034 | 0.978 | 0.001 | -0.998 | 0.001 | 1,1 |
| Fathead Minnow | -01.581 | 7.737 | -0.204 | 0.843 | 0.005 | -0.119 | 0.042 | 1,8 |
| Round Goby | 10.061 | 3.276 | 3.071 | 0.010 | 0.440 | 0.393 | 9.432 | 1,12 |
| Sand Shiner | -00.349 | 2.293 | -0.152 | 0.881 | 0.002 | -0.065 | 0.023 | 1,15 |
| Spotfin Shiner | 00.992 | 2.520 | 0.394 | 0.732 | 0.072 | -0.392 | 0.155 | 1,2 |
| White Sucker | 01.063 | 0.921 | 1.154 | 0.268 | 0.087 | 0.022 | 1.331 | 1,14 |
| Zoobenthivore | 04.709 | 1.778 | 2.648 | 0.014 | 0.219 | 0.188 | 7.014 | 1,25 |
| Omnivore | -00.740 | 1.689 | -0.438 | 0.665 | 0.007 | -0.029 | 0.192 | 1,28 |
| Detritivore | 01.117 | 0.897 | 1.246 | 0.232 | 0.094 | 0.033 | 1.553 | 1,15 |

Supplemental Table 4: Linear regression analyses for microplastic fiber length and fish body length. Fish were collected summer 2016 at Muskegon, Milwaukee, and St. Joseph Rivers. Analyses were conducted on fish taxa with *n* ≥ 3.

| **Taxa** | **Coeff.** | **Stand. Error** | **t-value** | ***P*-Value** | **Mult. R^2^** | **Adj. R^2^** | **F-Statistic** | **df** |
| --- | --- | --- | --- | --- | --- | --- | --- | --- |
| Pooled | 0.046 | 0.723 | 1.707 | 0.089 | 0.005 | -0.003 | 2.913 | 1,552 |
| Banded Killifish | 0.066 | 0.069 | 0.953 | 0.346 | 0.023 | -0.002 | 0.909 | 1,39 |
| Bass sp. | 0.363 | 0.265 | 1.370 | 0.179 | 0.048 | 0.023 | 1.878 | 1,37 |
| Emerald Shiner | -0.403 | 0.362 | -1.114 | 0.278 | 0.056 | 0.011 | 1.241 | 1,21 |
| Fathead Minnow | 0.484 | 0.493 | 0.982 | 0.333 | 0.028 | -0.001 | 0.964 | 1,33 |
| Round Goby | -0.003 | 0.072 | -0.037 | 0.970 | 9.049e-06 | -0.001 | 0.001 | 1,152 |
| Sand Shiner | 0.096 | 0.066 | 1.444 | 0.151 | 0.014 | 0.007 | 2.086 | 1,151 |
| Spotfin Shiner | 0.066 | 0.256 | 0.258 | 0.798 | 0.002 | -0.031 | 0.066 | 1,30 |
| Spottail Shiner | 0.992 | 3.269 | 0.303 | 0.764 | 0.004 | -0.041 | 0.092 | 1,22 |
| White Sucker | -0.005 | 0.035 | -0.143 | 0.887 | 0.000 | -0.023 | 0.020 | 1,42 |

Supplemental Table 5: Chi square test of independence of microplastic category, fiber color, and size class collected from the Muskegon, Milwaukee, and St. Joseph Rivers and from the coast of Maine.

| **Metric** | **Sample Type** | **df** | ***X^2^*** | ***P*-Value** |
| --- | --- | --- | --- | --- |
| Category | Surface Water | 3 | 13.44 | < 0.010 |
|  | Fish | 2 | 00.08 | 0.959 |
| Color | Surface Water | 6 | 66.05 | < 0.001 |
|  | Fish | 4 | 03.27 | 0.514 |
| Size Class | Surface Water | 6 | 68.10 | < 0.001 |
|  | Fish | 4 | 03.27 | 0.514 |

Supplemental Table 6: Chi square test of independence of microplastic category, fiber color, and size class pooled across sites compared to lab controls.

| **Sample Type** |  | **df** | ***X^2^*** | ***P*-Value** |
| --- | --- | --- | --- | --- |
| Category | Surface Water | 2 | 02.58 | 0.275 |
|  | Fish | 1 | 00.09 | 0.760 |
| Size | Surface Water | 2 | 10.35 | 0.006 |
|  | Fish | 2 | 05.25 | 0.073 |
| Color | Surface Water | 7 | 27.76 | 0.001 |
|  | Fish | 8 | 19.55 | 0.012 |

**Supplemental Figures Legends**

Supplemental Figure 1: Mean microplastic between fish FFG within river sites during summer 2016. NA indicates not applicable because there were no fish collected for that FFG within the indicated river site.

Supplemental Figure 2: Relative abundance of microplastic categories in surface water (a) and fish (b) collected from the Muskegon, Milwaukee, and St. Joseph Rivers. Maine coast refers to surface water microplastic data from Barrows et al. 2016 collected off the coast of Maine, USA. Other category refers to all non-fiber microplastic collected off the coast of Maine, USA.

Supplemental Figure 3: Relative abundance of fiber color in surface water (a) and fish (b) collected from the Muskegon, Milwaukee, and St. Joseph Rivers. Maine coast refers to surface water fiber color data from Barrows et al. 2016 collected off the coast of Maine USA. Other category refers to all fiber colors except blue or clear microplastic collected off the coast of Maine, USA.

**Supplemental Figures**

Supplemental Figure 1:


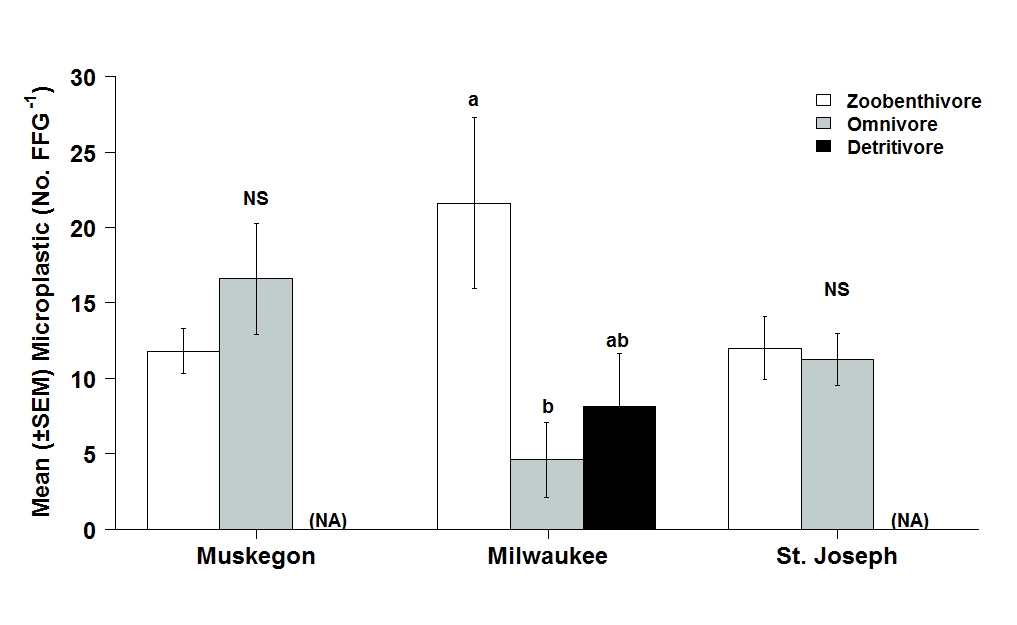


Supplemental Figure 2:


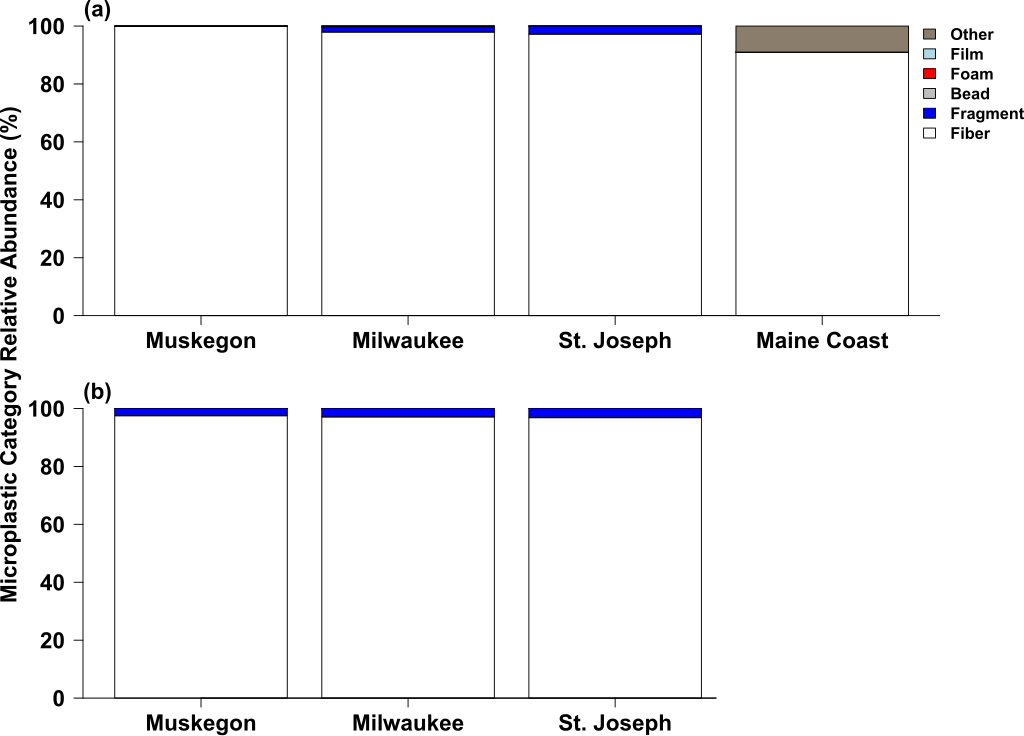


Supplemental Figure 3:


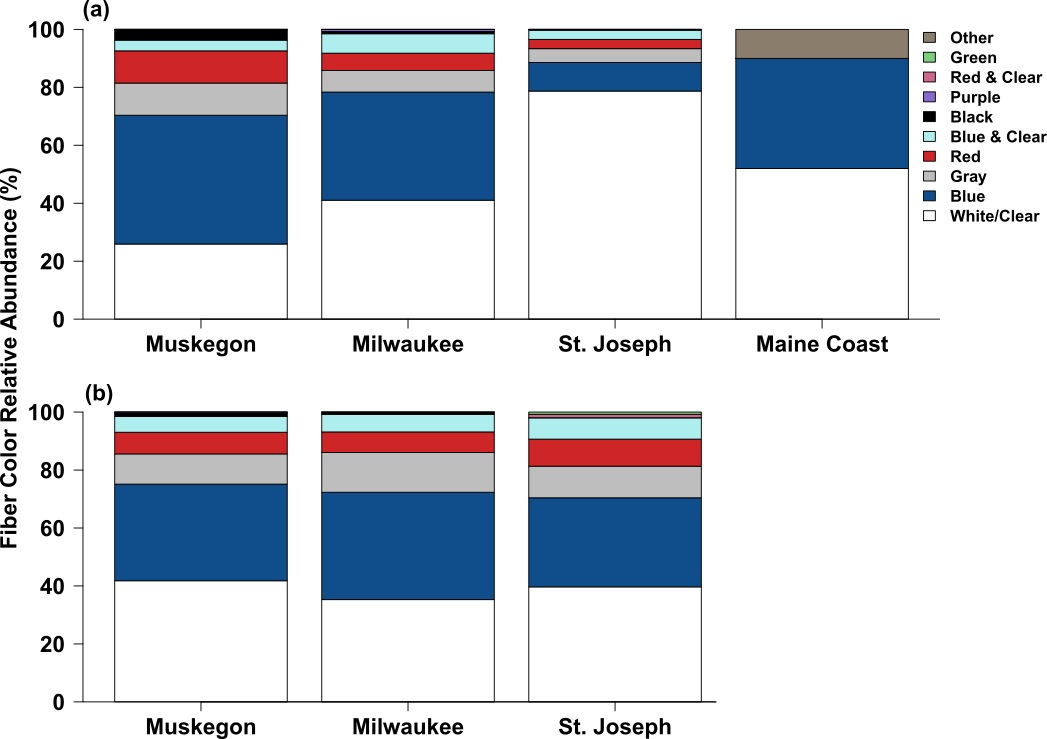

Supplement: Supplementary file 1 — Supplemental Materials [file 41598_2018_29980_MOESM1_ESM.doc]
